# MSCs Contribute to the Conversion of Ly6C^high^ Monocytes into Ly6C^low^ Subsets under AMI

**DOI:** 10.1155/2020/2460158

**Published:** 2020-01-13

**Authors:** Wenbin Lu, Genshan Ma, Zulong Sheng, Qingjie Wang, Lijuan Chen, Junhua Qi, Ronghui Shi, Jingjing Ji, Zhenjun Ji, Qiming Dai

**Affiliations:** ^1^Department of Cardiology, Zhongda Hospital Affiliated with Southeast University, China; ^2^Department of Cardiology, The Affiliated Changzhou No. 2 People's Hospital of Nanjing Medical University, China 213003; ^3^Department of Cardiology, Zhongda Hospital Affiliated with Southeast University, Lishui Branch, China

## Abstract

**Background:**

Ly6C^high^ monocytes are inflammatory cells that accumulate in an infarcted myocardium, and Ly6C^low^ monocytes are believed to be reparative and curb myocardial remodeling. NR4A1 is a novel target for modulating the inflammatory phenotype of monocytes during atherogenesis.

**Objectives:**

We aimed to investigate whether MSCs can contribute to the heterogeneity of Ly6C^high^ monocytes differentiated into Ly6C^low^ monocytes and whether this regulation is related to nuclear receptor NR4A1.

**Methods:**

Ly6C^high/low^ monocytes were first cocultured with MSCs. C57BL/6^CX3CR1-/-^ mice and C57BL/6 wild-type mice were then used to construct AMI models, and survival functions in the two groups were further compared. Ly6C^high/low^ monocytes in circulation and in MI tissue of C57BL/6^CX3CR1-/-^ AMI mice with or without MSC transplantation were determined by flow cytometry at day 1 and day 3. NR4A1 expression was further determined by Western blot. Apoptosis of cardiac myocytes in the infarct border zone at day 3 and day 7 was identified by TUNEL kits. Angiogenesis in the AMI heart at day 7 and day 21 was determined through immunohistochemistry by CD31.

**Results:**

We first demonstrated that the percentage of Ly6C^low^ monocytes increased greatly after 3 days of coculture with MSCs (12.8% ± 3.77% vs. 3.69% ± 0.74%, *p* < 0.001). The expression of NR4A1 in Ly6C^high/low^ monocytes was also significantly elevated at that time (1.81 ± 0.46 vs. 0.43 ± 0.09, *p* < 0.001). Following AMI, the percentage of circulating Ly6C^low^ monocytes in C57BL/6^CX3CR1-/-^ mice was significantly lower than that in C57BL/6 wild-type mice (4.36% ± 1.27% vs. 12.17% ± 3.81%, *p* < 0.001). The survival rate of C57BL/6^CX3CR1-/-^ mice (25%) was significantly lower than that of C57BL/6 wild-type mice (56.3%) after AMI (*χ*^2^ = 4.343, *p* = 0.037). After MSCs were transplanted, we observed a significant increase in Ly6C^low^ monocytes both in circulation (16.7% ± 3.67% vs. 3.22% ± 0.44%, *p* < 0.001) and in the MI heart (3.31% ± 0.69% vs. 0.42% ± 0.21%, *p* < 0.001) of C57BL/6^CX3CR1-/-^ mice. Western blot analysis further showed that the expression level of NR4A1 in the MI hearts of C57BL/6^CX3CR1-/-^ mice increased significantly under MSC transplantation (0.39 ± 0.10 vs. 0.11 ± 0.04, *p* < 0.001). We also found significantly decreased TUNEL^+^ cardiac myocytes (15.45% ± 4.42% vs. 22.78% ± 6.40%, *p* < 0.001) in mice with high expression levels of NR4A1 compared to mice with low expression levels. Meanwhile, we further identified increased capillary density in the infarct zones of mice with high expression levels of NR4A1 (0.193 ± 0.036 vs. 0.075 ± 0.019, *p* < 0.001) compared to mice with low expression levels 21 days after AMI.

**Conclusions:**

MSCs can control the heterogeneity of Ly6C^high^ monocyte differentiation into Ly6C^low^ monocytes and further reduce inflammation after AMI. The underlying mechanism might be that MSCs contribute to the increased expression of NR4A1 in Ly6C^high/low^ monocytes.

## 1. Introduction

Curbing myocardial remodeling after AMI remains a major challenge [1, 2]. Stem cell transplantation into the injured heart after AMI is still believed to reduce initial damage, promote activation of the regenerative potential of the heart, and integrate the regenerated tissue better into the organ. Mesenchymal stem cells (MSCs) have long been used as optimal stem cells that can be transplanted after AMI due to their unique characteristics of immune regulation and paracrine function [3–5]. Mesenchymal stem cells (MSCs) exhibit complex interactions with various immune cells, including monocytes and macrophages, which are believed to regulate the immune microenvironment during tissue repair and provide a good “soil” for tissue regeneration. MSCs adopt a specific phenotype to suppress or promote immune responses depending on the inflammatory microenvironment in which they reside. Current studies on the immunomodulatory abilities of MSCs have focused on the interaction between MSCs and inflammatory monocytes [6, 7]. Our previous studies have proven that a lower deployment of Ly6C^high^ monocytes after AMI could improve the effectiveness of MSC transplantation and selectively ameliorate myocardial remodeling [8].

The recent recognition of physiological and pathological deployment of Ly6C^high/low^ monocytes following AMI provides a fundamental basis for the treatment of inflammation [9, 10]. Proinflammatory Ly6C^high^ monocytes are predominant in the first few days following AMI and promote digestion of the infarcted tissue and necrotic debris, whereas reparative Ly6C^low^ monocytes predominate during the resolution of inflammation over the next few days and are believed to be atheroprotective [11]. In recent years, the effect of MSCs on monocytes has become increasingly clear. However, whether MSCs can reprogram monocytes from the inflammatory Ly6C^high^ phenotype to the anti-inflammatory Ly6C^low^ phenotype is yet to be determined. Whether MSCs within tissues can induce monocyte migration and convert them into a regulatory phenotype is also controversial.

Although MSCs have been found to promote repair, the mechanism of repair has not been clearly explained. Recent progress in understanding immunomodulatory inflammation in response to heart injuries drives the exploration of effective therapeutic approaches for AMI. As compared to C57BL/6 mice, C57BL/6^CX3CR1-/-^ mice were introduced in our experiments. C57BL/6^CX3CR1-/-^ mice lack the critical gene of CX3CR1 [12, 13] which mainly drives the employment of Ly6C^low^ monocytes in the spleen and bone marrow to circulation and infarcted myocardium when in AMI. As a result, using C57BL/6^CX3CR1-/-^ mice with a relatively low level of Ly6C^low^ monocytes, we can test effectively the patterns of Ly6C^high^ monocytes converted to Ly6C^low^ monocytes with or without MSCs. Meanwhile, NR4A1 is believed to be key regulators for Ly6C^low^ monocytes. Thus, the main objective of our study is to investigate whether the immunomodulatory function of MSCs is related to the heterogeneity of Ly6C^high^ monocytes differentiated into Ly6C^low^ monocytes and the role of nuclear receptor NR4A1 during the process.

## 2. Materials and Methods

### 2.1. MSC Preparation and Labeling

Mouse-derived MSCs (from C57BL/6 wild-type mice) were prepared as previously described [14]. The femurs and tibias of 6-week-old mice were first flushed with PBS (HyClone) to collect the bone marrow cells. After centrifugation at 1000 rpm/min for 5 min, the cells were washed 3 times and then resuspended in minimal essential medium supplemented with 10% FBS (DMEM, Gibco) at a density of 5 × 10^6^ cells/cm^2^. Nonadherent cells were removed at day 3. The cells were then selected and identified by the presence of surface markers through flow cytometry (Becton Dickinson Inc., Franklin Lakes, NJ, USA) as previously described [15] (positive for Sca-1, CD105, and CD90 and negative for CD45, CD34, and CD31). The 3^rd^ passage of cells was used for subsequent experiments.

### 2.2. Isolation of Monocytes and Coculture

The monocytes were obtained as follows: First, 6-week-old C57BL/6 mice were sacrificed. The skin of the belly was then opened by pulling the skin apart on the left side of the abdomen, leaving the abdominal membrane intact until the spleen was excised. Single-cell suspensions were obtained from the spleen by digestion with a cocktail of 450 U/ml collagenase II, 125 U/ml collagenase IV, and 60 U/ml hyaluronidase (Sigma-Aldrich) for 30 min at 37°C with shaking. After filtration through a 200-mesh screen and centrifugation at 400 g, the cells were positively selected using CD11b by flow cytometry (Thermo Fisher Scientific, CN). The purified cells were cultured in RPMI medium 1640 supplemented with 10% FCS in 6-well plates. The monocytes used for the experiments were from the 3^rd^ passage. Transwell chambers (8.0 *μ*m pore size, Millipore) were used to examine the interactions between the monocytes and MSCs obtained above (coculture for 24-72 h).

### 2.3. Mouse Care and AMI Model

All animal experiments performed in this study were approved by the Institutional Animal Care and Use Committee (IACUC) of Southeast University (20180828003). All procedures followed were in accordance with the ethical standards of the responsible committee on animal experimentation (institutional and national) and with the Helsinki Declaration revised in 1975. All procedures on mice were performed in compliance with National Institutes of Health and IACUC guidelines. C57BL/6 and C57BL/6^CX3CR1-/-^ mice (purchased from Beijing Biocytogen Co., Ltd.) were fed a regular diet and placed in a dedicated room (25°C-28°C).

AMI mice were induced by direct ligation of the left anterior descending artery. Male or female (paired) mice (6–8 weeks old) weighing 20–25 g were used in the experiment. After being anesthetized (0.1% pentobarbital sodium (50 mg/kg)) and under ventilator-assisted breathing (110 breaths/minute; Harvard Apparatus), approximately 1 cm of the wound layer was cut along the 3–4 intercostal space of the left chest. After separation of the pericardium, the left side branch of the coronary artery was ligated with an 8–0 nylon suture below the left auricular level. ST elevation at three unipolar limb leads (left upper limb and two lower limbs) was considered to be a successful AMI model.

### 2.4. Flow Cytometric Analysis and Western Blots

Monocytes were isolated by gradient density centrifugation following erythrocyte lysis. After washing twice with PBS (4°C), the cells were resuspended at a concentration of 10^6^ cells/ml. Single-cell suspensions were then incubated with FITC-rat anti-mouse CD11b and PE-rat anti-mouse Ly6C (BD Biosciences). CD11b^+^Ly6C^high^ monocytes were further identified using a flow cytometer (Thermo Fisher Scientific, CN). Briefly, CD11b monocytes were gated in a side scatter; subsequently, subpopulations of Ly6C^high^ and Ly6C^low^ monocytes were distinguished by their surface expression pattern of Ly6C.

Monocytes or infarcted myocardium was washed twice with PBS, lysed in lysis buffer (10 mmol/l Tris-HCl, pH 7.4, containing 1% Triton X-100, 100 mmol/l sodium chloride, 20 mmol/l sodium pyrophosphate, 2 mmol/l sodium orthovanadate, 2 mmol/l EDTA, and 1% protease inhibitor cocktail (Sigma)), and centrifuged at 12,000 g for 30 min at 4°C. The protein concentration was determined using a protein assay kit (Thermo Scientific, USA), and the samples were mixed with SDS-denaturing sample buffer and separated on 10% SDS-PAGE gels. The proteins were transferred to a PVDF membrane by electrophoresis. The membrane was blocked and incubated overnight on a rocking platform at 4°C with antibodies against NR4A1 (Santa Cruz Biotechnology) and GAPDH (1 : 1000; Kangchen Biology Inc., China). The membranes were then incubated with HRP-conjugated secondary antibodies for 1 h and exposed using a Molecular Imager ChemiDoc XRS System (Bio-Rad). The relative intensities of the protein bands were analyzed using Image-Pro Plus 6.0 (Media Cybernetics, Silver Spring, MD, USA).

### 2.5. Immunostaining

The animals were sacrificed on specific days following AMI. The tissues were fixed in formalin and embedded in paraffin blocks according to established protocols. The fixed hearts were serially cut 8 *μ*m from the apex to the level just below the coronary artery ligation site. After antigen retrieval, the specimens were incubated with 1% normal blocking serum in PBS for 60 min to suppress the nonspecific binding of IgG. The slides were then incubated for 30-60 min with each mouse antibody or fluorescent reagent. A TUNEL kit (R&D Systems) was used to identify the nuclei of the apoptotic cardiac myocytes in the infarct border zone on days 3 and 7. CD31 (platelet endothelial cell adhesion molecule-1, PECAM-1/CD31, BD Biosciences) was used for immunohistochemistry to measure vessel density at day 3 and day 21. Microscopy (Olympus BX61) was used as necessary to obtain images of the immunostaining results.

### 2.6. Statistical Analysis

SPSS 23.0 was used for data analysis and comparison between groups in the experiment. Depending on the expression and distribution of data, an independent *t*-test (for normally distributed data and continuous data) and a chi-squared test or Fisher's exact test (for categorical variable data) were used. Kaplan-Meier survival curves were used to compare event-free survival of the mice in the two groups. All the results in the experiment were considered to be statistically significant when the two-sided *p* value was ≤0.05.

## 3. Results

### 3.1. Conversion of Ly6C^high^ to Ly6C^low^ Monocytes When Cocultured with or without MSCs

**Figure 1:**
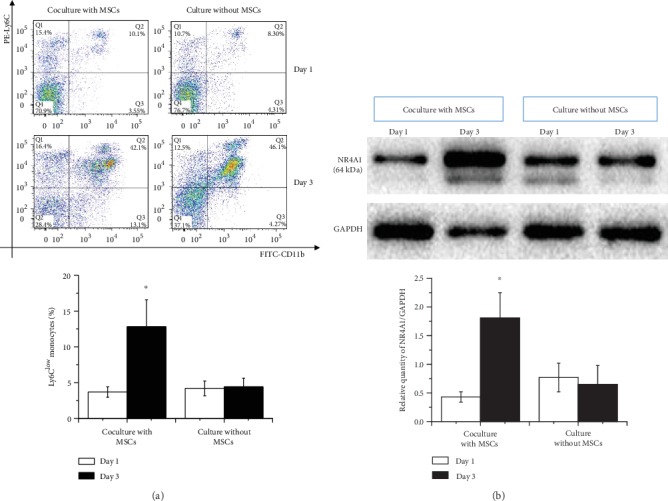
Conversion of Ly6C^high^ to Ly6C^low^ monocytes when cocultured with MSCs. (a) After stimulation with LPS (1 *μ*g/ml), monocytes were cocultured with or without MSCs (control). Ly6C^low^ monocytes at day 1 and day 3 were then determined by FACS. (b) Expression levels of NR4A1 in two groups of Ly6C^high/low^ monocytes (corresponding to (a)) were then tested. ^∗^*p* < 0.05 at day 3 vs. the first day. Abbreviations: FITC: fluorescein isothiocyanate; MSCs: mesenchymal stem cells; PE: phycoerythrin; NR4A1: nuclear receptor subfamily 4 group A member 1.

MSCs have long been known as stem cells with immunomodulatory functions. Ly6C^high/low^ monocytes were first cocultured with MSCs in a transwell chamber. We first made the proportion of Ly6C^low^ monocytes in two groups that were comparable (day 1) when cultured with and without MSCs (3.69% ± 0.74% vs. 4.20% ± 1.03%, *p* = 0.32). As expected, 3 days later, we found a great increase of Ly6C^low^ in the group being cocultured with MSCs (12.8% ± 3.77% vs. 3.69% ± 0.74%, *p* < 0.001). And no significant difference was shown in the corresponding control group without MSCs (4.41% ± 1.22% vs. 4.20% ± 1.03%, *p* = 0.83) (Figure 1(a)). At the time, the expression levels of NR4A1 in these Ly6C^high/low^ monocytes were then tested. Similar to the trend of Ly6C^low^ monocytes, the expression level of NR4A1 significantly increased after 3 days of coculture with MSCs (1.81 ± 0.46 vs. 0.43 ± 0.09, *p* < 0.001) but not in the group without MSCs (0.65 ± 0.33 vs. 0.77 ± 0.25, *p* = 0.249) (Figure 1(b)).

### 3.2. Patterns of Circulating Ly6C^high/low^ Monocytes in AMI Mice Changed by MSCs

**Figure 2:**
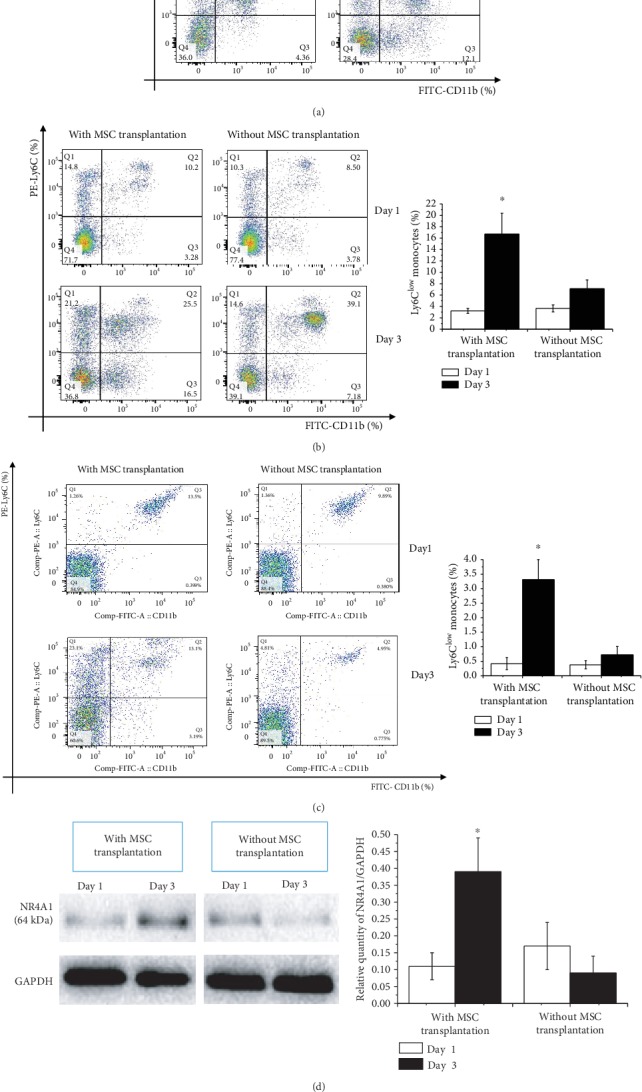
Conversion of Ly6C^high^ to Ly6C^low^ monocytes in AMI mice following transplantation with MSCs. (a) Ly6C^low^ monocyte levels in the circulation of AMI C57BL/6^CX3CR1-/-^ and AMI C57BL/6 AMI mice day 3. (b) Circulating Ly6C^low^ monocytes in AMI C57BL/6^CX3CR1-/-^ mice with or without MSC transplantation on day 1 and day 3. (c) Levels of Ly6C^low^ monocytes infiltrated in the AMI hearts of C57BL/6^CX3CR1-/-^ mice with or without MSC transplantation on day 1and day 3. (d) Levels of NR4A1 in AMI hearts of C57BL/6^CX3CR1-/-^ mice were then tested. ^∗^*p* < 0.05 at day 3 vs. the first day. Abbreviations: FITC: fluorescein isothiocyanate; MSCs: mesenchymal stem cells; PE: phycoerythrin; NR4A1: nuclear receptor subfamily 4 group A member 1; CX3CR1: C-X3-C motif chemokine receptor 1.

To further determine the immunomodulatory function of MSCs on Ly6C^high/low^ monocytes in vivo, C57BL/6^CX3CR1-/-^ mice (*n* = 12) were introduced and subjected to AMI. We first confirmed a relatively low level of Ly6C^low^ monocytes in the circulation at day 3 compared to the proportion of cells in C57BL/6 wild-type AMI mice (*n* = 12) (4.36% ± 1.27% vs. 12.17% ± 3.81%, *p* < 0.001) (Figure 2(a)). As expected, when MSCs were administered to the myocardium of the AMI heart of C57BL/6^CX3CR1-/-^ mice, we detected a greatly increased circulating Ly6C^low^ monocytes at day 3 (16.7% ± 3.67% vs. 3.22% ± 0.44%, *p* < 0.001) as compared to the C57BL/6^CX3CR1-/-^ mice without MSC transplantation (7.10% ± 1.55% vs. 3.66% ± 0.61%, *p* = 0.091) (Figure 2(b)).

As it is well known that inflammatory monocytes that infiltrated in the myocardium of AMI hearts are most critical to myocardial remodeling, we next set out to determine the patterns of Ly6C^high/low^ monocytes in the infarcted myocardium of C57BL/6^CX3CR1-/-^ mice with or without MSC transplantation. The results further confirmed a great increase in Ly6C^low^ monocytes after MSC infusion in the AMI hearts at day 3 (3.31% ± 0.69% vs. 0.42% ± 0.21%, *p* < 0.001) as compared to the control without given MSCs (0.72% ± 0.29% vs. 0.38% ± 0.14%, *p* = 0.244) (Figure 2(c)). Expression levels of NR4A1 in the MI hearts of C57BL/6^CX3CR1-/-^ mice were further tested; similar to the expression patterns of Ly6C^low^ monocytes shown above, the expression levels of NR4A1 in C57BL/6^CX3CR1-/-^ AMI mouse hearts increased significantly at day 3 under MSC transplantation (0.39 ± 0.10 vs. 0.11 ± 0.04, *p* < 0.001) compared to the group without MSC transfusion (0.09 ± 0.05 vs. 0.17 ± 0.07, *p* = 0.079) (Figure 2(d)).

### 3.3. C57BL/6^CX3CR1-/-^ Mice Showed Decreased Survival Functions after AMI

**Figure 3:**
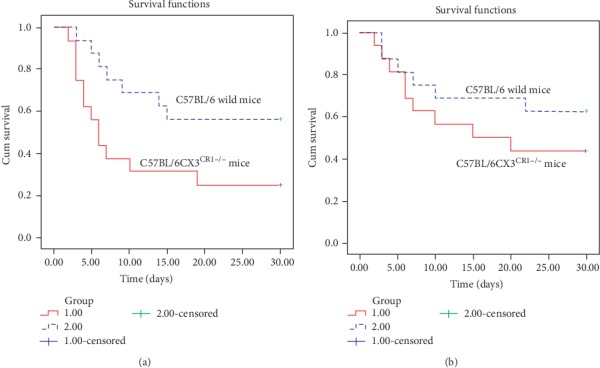
Univariate Kaplan-Meier analysis. (a) Survival functions of C57BL/6^CX3CR1-/-^ mice (red solid line) and C57BL/6 wild mice (blue dotted line) without MSC transplantation after AMI. (b) Survival functions of C57BL/6^CX3CR1-/-^ mice (red solid line) and C57BL/6 wild mice (blue dotted line) following MSC transplantation after AMI. Abbreviations: MSCs: mesenchymal stem cells.

To further determine whether the number of Ly6C^low^ monocytes affected the survival of mice after AMI, survival functions at 30 days were first compared in C57BL/6^CX3CR1-/-^ mice and C57BL/6 wild-type mice (*n* = 16 in each group) with or without MSC transplantation. The results showed that the survival rate of C57BL/6^CX3CR1-/-^ mice (25%) was significantly lower than that of C57BL/6 wild-type mice (56.3%) without MSC infusion after AMI (*χ*^2^ = 4.343, *p* = 0.037) (Figure 3(a)), which directly indicated the importance of Ly6C^low^ monocytes on prognosis. However, when MSCs were transplanted (*n* = 16 in each group), we found that the survival rates in C57BL/6^CX3CR1-/-^ mice (43.8%) and C57BL/6 wild-type mice (62.5%) were comparable (*χ*^2^ = 1.089, *p* = 0.297) (Figure 3(b)). Though the results might be related to a very small sample size, they directly suggest that MSCs promoting monocyte phenotypic transition played a very important role in this process.

### 3.4. Decreased Apoptosis of Cardiac Myocytes under the Circumstance of Elevated NR4A1 in AMI Heart

**Figure 4:**
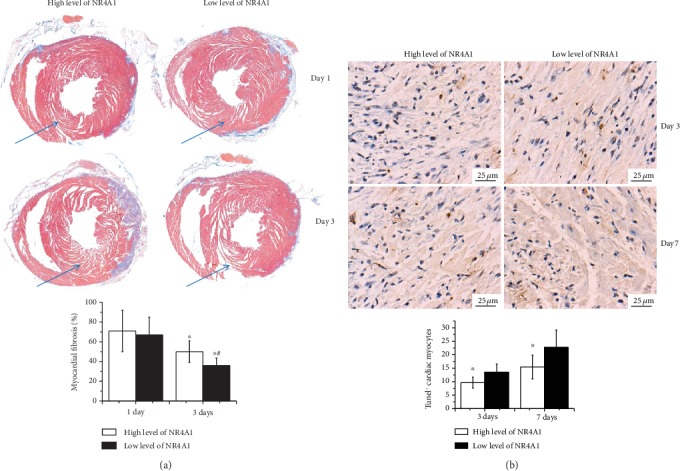
Myocardial remodeling and apoptosis of cardiac myocytes under different levels of NR4A1. (a) Direct visualization of the lysis of myocardial fibrosis after myocardial infarction through Mason staining at day 1 and day 3. ^∗^*p* < 0.05 vs. day 1, ^#^*p* < 0.05 vs. the corresponding low-NR4A1 expression level group; (b) TUNEL^+^ cardiac myocytes at day 3 and day 7 were then separated in two groups as defined above. ^∗^*p* < 0.05 vs. the corresponding low-NR4A1 expression level group. Abbreviations: NR4A1: nuclear receptor subfamily 4 group A member 1; TUNEL: terminal deoxynucleotidyl transferase-mediated dUTP nick-end labeling.

Here, we have shown that MSCs contribute to the transition of circulating Ly6C^high^ monocytes into Ly6C^low^ monocytes, which is always accompanied with an increased expression level of NR4A1. We next wondered whether the potential mechanism of decreased myocardial remodeling is related to NR4A1. Based on the different expression levels of NR4A1 identified by Western blot in the AMI heart of C57BL/6^CX3CR1-/-^ mice after MSC transfusion (NR4A1/GAPDH ≥ 0.2), we first found an obvious trend of myocardial fiber lysis and decrease in low-level NR4A1 group through Masson staining at day 3 after AMI (36% ± 7.5% vs. 50% ± 11%, *p* < 0.001) (Figure 4(a)), though there is no significant difference concerning thickness of the left ventricular wall at the time (data not shown). Under this circumstance, we next detected the apoptosis of cardiac myocytes under these conditions (*n* = 12 in each group). The results confirmed that both at day 3 (9.66% ± 2.04% vs. 13.54% ± 3.01%, *p* < 0.001) and at day 7 (15.45% ± 4.42% vs. 22.78% ± 6.40%, *p* < 0.001), counts of TUNEL^+^ cardiac myocytes were significantly reduced in the high-NR4A1 expression level hearts compared to the low-level group (Figure 4(b)).

### 3.5. Increased Angiogenesis Was Observed under Elevated NR4A1 Levels in the AMI Heart

**Figure 5:**
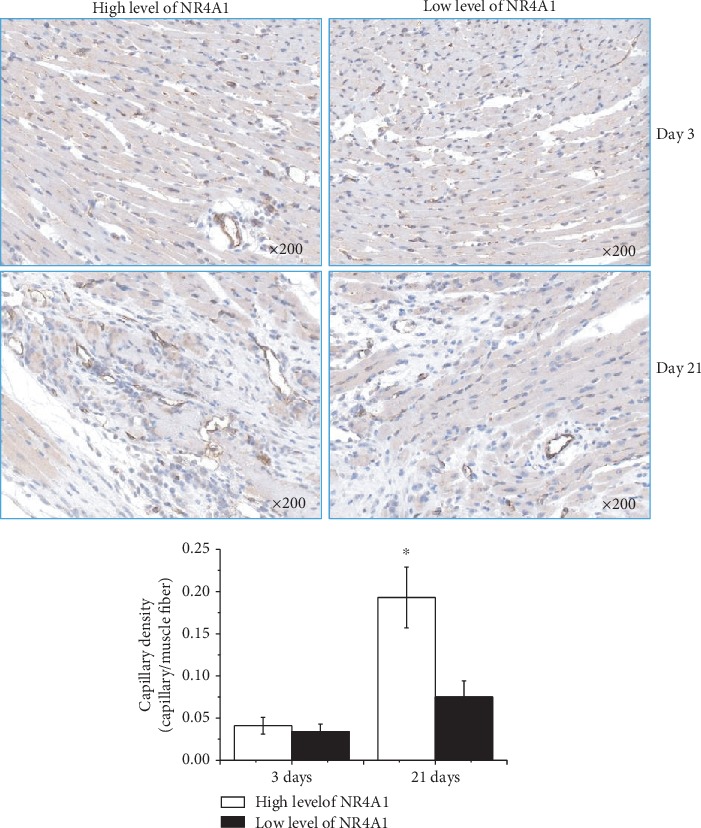
IHC analysis for the expression of CD31 in AMI mouse hearts of different groups (magnification ×200). CD31-positive endothelial cells in C57BL/6^CX3CR1-/-^ AMI mice following MSC transplantation were compared on days 3 and 21. ^∗^*p* < 0.05 vs. the corresponding low-NR4A1 expression level group. Abbreviations: NR4A1: nuclear receptor subfamily 4 group A member 1.

Having found decreased apoptosis of cardiomyocytes in MI hearts of C57BL/6^CX3CR1-/-^ mice with elevated expression levels of NR4A1, we next intended to test whether the elevated NR4A1 levels can contribute to neovascularization after AMI. The expression of CD31-positive endothelial cells at day 3 and day 21 was detected (*n* = 6 in each group). IHC analysis confirmed that CD31-positive endothelial cells were comparable at day 3 (0.041 ± 0.010 vs. 0.034 ± 0.009, *P* = NS. However, we further found that CD31-positive endothelial cells in the elevated NR4A1 group increased greatly at day 21 (0.193 ± 0.036 vs. 0.075 ± 0.019, *p* < 0.001) (Figure 5(a)), which indicated a very positive role of NR4A1 for myocardial repair after MI.

## 4. Discussion

Studies have gradually confirmed that monocytes, vespecially Ly6C^high^ subpopulations and their derived inflammatory mediators, are important factors leading to cardiomyocyte necrosis and the progression of remodeling after a myocardial infarction. On the other hand, the Ly6C^low^ subsets promoting the subsiding of inflammation play an important role in clearing necrotic cellular debris and maintaining vessel wall stability in the next phase after myocardial infarction. Thus, controlling the balance and infiltration of inflammatory cells and promoting the clearance and regression of inflammatory mediators as early as possible will probably extenuate myocardial remodeling after MI and improve prognosis. Due to their superior biological characteristics as previously described [16, 17], MSCs have been extensively investigated as a cell-based therapy for cardiac repair. Here, we found a new feature of MSCs in ameliorating the prognosis of myocardial infarction by transforming Ly6C^high^ monocytes into Ly6C^low^ monocytes as well as the underlying mechanisms of this amelioration.

By using a coculture model and C57BL/6^CX3CR1-/-^ mice to test the accumulation and distribution of Ly6C^high/low^ monocytes post-AMI and the subsequent expression changes of NR4A1 in monocytes and the myocardium, this study demonstrated the following: (1) MSCs are immunomodulatory cells that can contribute to the differentiation of Ly6C^high^-phenotype monocytes into Ly6C^low^-phenotype monocytes; (2) a relatively and extremely low level of Ly6C^low^-phonotype monocytes exists in the circulation of C57BL/6^CX3CR1-/-^ mice even after AMI; (3) under the systemic transplantation of MSCs, obviously increased proportions of Ly6C^low^-phonotype monocytes were observed in C57BL/6^CX3CR1-/-^ mice, as evidenced by flow cytometric analysis; and (4) an increased expression of Ly6C^low^ monocytes following MSC transplantation was always accompanied by an increase in expression levels of NR4A1. The reduced apoptosis of cardiomyocytes three days after AMI and the increased regeneration of capillaries at three weeks further indicated that nuclear receptor NR4A1 may play an important role in the process of monocyte phenotype transformation.

Ly6C^low^ monocytes, also called patrolling monocytes, act as scavengers and patrol the vessel wall for surveillance. Previous reports have shown that Ly6C^low^ monocytes from the spleen infiltrate the ischemic heart as a second phase of immune response (following Ly6C^high^ monocyte infiltration) and promote inflammation reduction. The CX3CL1/CX3CR1 axis is known to be a key regulator of the recruitment and infiltration of Ly6C^low^ monocytes in the ischemic heart, as patrolling monocytes express CX3CR1 at much higher levels. Studies have demonstrated that CX3CR1 deletion reduces the patrolling and number of Ly6C^low^ monocytes [18].

Consequently, in the present study, after coculturing MSCs and monocytes, we further detected the heterogeneity of Ly6C^high^ and Ly6C^low^ monocytes in vivo using C57BL/6^CX3CR1-/-^ mice, which have a relatively low level of Ly6C^low^ monocytes compared to the C57BL/6 wild-type mice. Additionally, we confirmed that the survival rates of C57BL/6^CX3CR1-/-^ mice were significantly lower than those of C57BL/6 wild-type mice after AMI. Therefore, only in this case can we achieve the most realistic phenotypic transformation of monocytes by MSCs. Subsequently, we further confirmed our hypothesis that MSC transplantation significantly increased the expression of Ly6C^low^ monocytes and reduced the mortality of C57BL/6^CX3CR1-/-^ mice after AMI. Of course, in this process, we observed a relatively low level of (but not zero) patrolling monocytes in the circulation of C57BL/6^CX3CR1-/-^ mice, which is likely because in addition to CX3CR1, CCR5 expression in monocytes is at least partially responsible for the recruitment of patrolling monocytes, even at low levels [19–21].

The mechanisms underlying how MSCs can control the heterogeneity of Ly6C^high^ monocyte differentiation into Ly6C^low^ monocytes have always been a critical issue that has lacked explanation. Recent studies have identified NR4A1 as a key molecular signal that is critical for driving the differentiation of subsets of Ly6C^low^ [22, 23]. Researchers have previously shown that deleting an Nr4a1 superenhancer subdomain could ablate Ly6C^low^ monocyte production, which fully suggests that the orphan nuclear receptor NR4A1 has the ability to suppress inflammatory activity and is involved in the regulation of patrolling monocytes [24]. As a result, we next determined whether the heterogeneity of Ly6C^high^ monocyte differentiation into subsets of Ly6C^low^ by MSCs is related to the nuclear receptor NR4A1; our results indicated that the patterns of NR4A1 expression level increase were similar to those of Ly6C^low^ monocytes. According to previous studies [25, 26] and our research, we believe that the reason for this relationship is that nuclear receptor NR4A1 contributes to the phenotypic transformation of monocytes.

Our previous research had preliminarily proven that MSC transplantation curbed myocardial remodeling [11]; however, although higher secretions of growth factors were identified as an underlying mechanism, other factors may also be involved. The anti-inflammatory properties of NR4A1 indicate that during the process of monocyte regulation by MSCs, it might be a protective reaction referred to as resolving inflammation. Therefore, we next detected the apoptosis of cardiac myocytes and neovascularization after AMI under MSC transplantation according to the different expression levels of NR4A1 in the MI heart. The results further proved that TUNEL^+^ cardiac myocytes were significantly reduced when at the high-NR4A1 expression level, and the number of CD31-positive endothelial cells was also greatly increased. In conclusion, both of these results suggest that NR4A1/Nur77 is a novel target for modulating inflammation and is also important for the repression of myocardial remodeling.

## 5. Conclusion

By using a coculture model and C57BL/6^CX3CR1-/-^ mice to test the accumulation and distribution of Ly6C^high/low^ monocytes post-AMI and the subsequent expression changes of NR4A1 in Ly6C^high/low^ monocytes and MI myocardium, the present study demonstrated that MSCs can control the heterogeneity of Ly6C^high^ monocyte differentiation into Ly6C^low^ monocytes. By further grouping the mice according to the level of NR4A1 expression following AMI, we demonstrated the underlying mechanisms through which MSCs might contribute to the increased expression of NR4A1 in Ly6C^high/low^ monocytes. NR4A1 showed great anti-inflammatory properties during the process of monocyte phenotype transformation driven by MSCs.

## Data Availability

The data that support the findings of this study are available from the corresponding authors upon reasonable request.

## Abbreviations

AMI: Acute myocardial infarction
GAPDH: Glyceraldehyde-3-phosphate dehydrogenase
CCR5: CC-chemokine receptor 5
CX3CL1: Fractalkine
CX3CR1: Chemokine (C-X3-C motif) receptor 1
MI: Myocardial infarction
MSCs: Mesenchymal stem cells
NR4A1: Nuclear receptor subfamily 4 group A member 1
TUNEL: TdT-mediated dUTP nick-end labeling.
